# Editorial: Exploring immunological factors in inner ear disorders: allergy and inflammation

**DOI:** 10.3389/fimmu.2026.1958202

**Published:** 2026-08-18

**Authors:** Agnieszka J. Szczepek, Vincent Van Rompaey, Bohua Hu

**Affiliations:** 1 Department of Otorhinolaryngology, Head and Neck Surgery, Charité–Universitätsmedizin Berlin, Corporate Member of Freie Universität Berlin and Humboldt-Universität zu Berlin, Berlin, Germany; 2 Faculty of Medicine and Health Sciences, University of Zielona Góra, Zielona Góra, Poland; 3 Department Translational Neurosciences, Faculty of Medicine and Health Sciences, University of Antwerp, Antwerp, Belgium; 4 Department Otorhinolaryngology and Head and Neck Surgery, Antwerp University Hospital, Antwerp, Belgium; 5 Department of Speech, Language, and Hearing, The University of Texas at Dallas, Dallas, TX, United States

**Keywords:** cochlea, cochlear implant, inflammation, macrophages, presbycusis

For much of the twentieth century, the inner ear was imagined as a sanctuary: a delicate structure sealed away behind barriers, immune-privileged, and largely indifferent to the immune life of the body (1, 2). That picture has quietly come apart. We now understand that the cochlea contains its own resident immune cells, primarily macrophages (3, 4), and that inflammation plays a key role in almost all major acquired hearing and balance disorders (5). These include conditions caused by noise exposure, aging, ototoxicity, device implantation, and idiopathic diseases like Ménière’s disease. The sanctuary, it turns out, was never as isolated as once thought.

**Figure 1:**
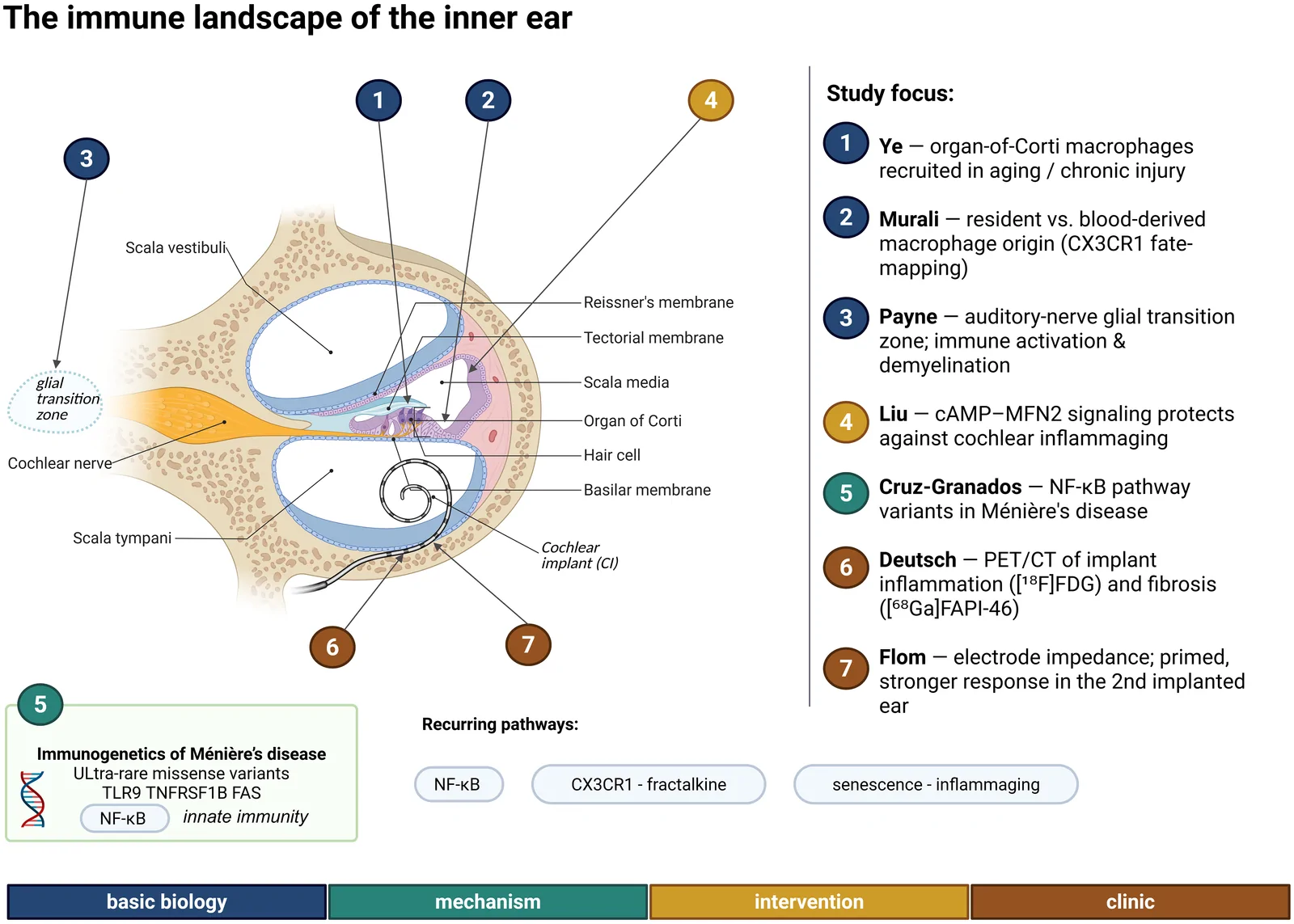
The immune landscape of the cochlea. Schematic mid-modiolar cross-section of one cochlear turn, mapping the seven contributions of this Research Topic onto the structures they address, from the organ of Corti and the auditory-nerve glial transition zone to the spiral ganglion and the cochlear-implant electrode interface. Callout colors denote position along the translational arc (basic biology, mechanism, intervention, clinic); recurring immune pathways (NF-κB, CX3CR1/fractalkine, senescence-associated inflammation) are noted below. Created with BioRender.com.

This Research Topic takes that shift in understanding as its starting point. The seven original research articles gathered here trace immune involvement across scales, from individual cell populations in the organ of Corti, through mechanisms of inflammatory and age-related degeneration, to the genetics of disease and the immune response at the electrode-tissue interface (**Figure 1**). Allergy, named in our call, ultimately went unaddressed by the contributions we received: whether this reflects a quiet field or simply that such work needs more time than a single submission window allows, we cannot say. What did arrive, however, tells us a remarkably coherent story.

It begins with the cells themselves and a question that has lingered for years: are there macrophages in the organ of Corti at all? Ye et al. answer with unusual clarity. Using a cyclodextrin ototoxicity model alongside aging cochleae, they show that the organ of Corti is free of macrophages under normal conditions and even during acute injury, but that these cells arrive during chronic pathology and aging. Interestingly, their recruitment aligns with supporting-cell pathology rather than sensory hair-cell loss, portraying macrophages as involved in later remodeling rather than as initial responders to damage.

If Ye et al. establish *when* these cells appear, Murali et al. ask *where* they come from. Using inducible CX3CR1 fate mapping in a model of acoustic trauma, they distinguish long-lived resident macrophages from blood-derived monocytes and macrophages and track their turnover, distribution, and fate. The distinction is more than bookkeeping: cells that look alike under the microscope may perform very different functions — including sustaining the survival of spiral ganglion neurons.

Payne et al. then widen the frame beyond the sensory epithelium to the auditory-nerve glial transition zone, the boundary within the modiolus where central and peripheral myelination meet. Combining tissue clearing, light-sheet microscopy, high-resolution 3D imaging, and RNA sequencing in a model of age-related hearing loss, they identify this niche as a focal point of immune activation and glial disruption in the aging ear — drawing a line from neuroimmune interaction to myelin breakdown and the decline of the auditory nerve.

With the landscape mapped, our Research Topic turns from description to intervention. Liu et al. move past cataloging inflammation and senescence to ask whether they can be reversed. In a D-galactose senescence model and in aged mice, boosting cyclic AMP signaling with dibutyryl-cAMP reduced cellular senescence and inflammation, restored mitochondrial function, and, given systemically, helped preserve hearing and protect hair cells. The effect leaned heavily on Mitofusin-2, pointing to a cAMP–MFN2 axis as a candidate therapeutic target in presbycusis, and offering a proof of principle that the cochlear inflammaging might be met pharmacologically rather than merely observed.

Cruz-Granados et al. go to the level of the genome, taking up the immune dysregulation that shadows many cases of Ménière’s disease. Mining exome data, they surface ultra-rare missense variants in NF-κB pathway genes — TLR9, TNFRSF1B, and FAS — and, through protein modeling, docking, and splicing analyses, argue that these variants destabilize their proteins and likely impair innate immune signaling. It is a mechanistic account of the autoinflammatory features seen in a subset of patients, grounding a clinical phenotype in specific molecular faults.

The final two studies carry the story to the preclinical and clinical levels, where an implanted electrode becomes a test of the ear’s own immunity. Deutsch et al. offer a PET/CT pilot in guinea pigs, using [18F]FDG to measure inflammation and [68Ga]FAPI-46 to track fibrosis around the electrode. They record rising inflammation after implantation and heightened fibrosis-related uptake a year later and — mindful of clinical scanner resolution — sketch a path toward imaging that could one day monitor how patients respond to anti-inflammatory or anti-fibrotic therapy. Flom et al. read the same foreign-body response through a different measure: electrode impedance, assessed in a retrospective cohort of bilaterally implanted patients. Impedance rose faster and higher in the second ear to be implanted — a signature of a primed, more vigorous response, and a hint that immunological memory itself may shape how the body receives a cochlear implant.

Read together, these seven papers converge on a handful of subjects. The first is heterogeneity: cochlear macrophages cannot be treated as a single entity, because their origin, activation state, location, and temporal context all shape what they do; misreading that diversity risks misreading their function. The second is agency: inflammation and senescence are not merely companions of degeneration but points of leverage, whether through the cAMP–MFN2 pathway, anti-fibrotic strategies, or management of the foreign-body response. A third is convergence: recurring pathways such as NF-κB signaling, fractalkine (CX3CR1) signaling, and senescence-driven inflammation surface across conditions that look, clinically, quite distinct, hinting at shared machinery beneath. Running through all of it is the full translational arc: from fate mapping in transgenic mice to human exome analysis and bedside impedance readings, the range this field requires to move from mechanism to medical intervention.

The gaps in current knowledge are as revealing as the actual findings. Much mechanistic research still depends on rodent models and needs validation using human temporal bone and single-cell data; immune phenotyping is not yet standardized across labs; and moving from potential targets or biomarkers to actual therapies or clinical tools remains an ongoing challenge. We hope this Research Topic helps in establishing immunology as a fundamental field within otology and fosters collaboration among imaging, genetics, and cell biology to turn these mechanisms into practical diagnostics and treatments.

We thank all the authors for their contributions and the reviewers whose careful work strengthened these papers. To those whose submissions could not be included, we offer encouragement: use the reviewers’ constructive comments to sharpen the work, and consider submitting to a future Research Topic in this growing field.
